# Impact of self-rated osteoarthritis severity in an employed population: Cross-sectional analysis of data from the national health and wellness survey

**DOI:** 10.1186/1477-7525-10-30

**Published:** 2012-03-15

**Authors:** Marco daCosta DiBonaventura, Shaloo Gupta, Margaret McDonald, Alesia Sadosky, Dan Pettitt, Stuart Silverman

**Affiliations:** 1 Kantar Health, New York, NY, USA; 2 Pfizer Inc., 235 East 42nd Street, New York, NY 10017, USA; 3 Cedars-Sinai Medical Center, Los Angeles, CA, USA

**Keywords:** Osteoarthritis, Burden, Workforce, Productivity, Quality of life

## Abstract

**Background:**

Although osteoarthritis (OA) often affects older persons, it has a profound effect on individuals actively employed. Despite reports of reduced productivity among workers with OA, data are limited regarding the impact of OA among workers. The objective of this study was to evaluate the impact of self-rated OA severity on quality of life, healthcare resource utilization, productivity and costs in an employed population relative to employed individuals without OA.

**Methods:**

This cross-sectional analysis used data derived from the 2009 National Health and Wellness Survey (NHWS). Multivariable analyses characterized outcomes and costs (direct medical costs and indirect) among workers (full-time, part-time, or self-employed) ≥ 20 years of age who were diagnosed with OA and who self-rated their OA severity as mild, moderate, or severe relative to workers without OA. Evaluated outcomes included productivity, assessed using the Work Productivity and Impairment (WPAI) scale; health-related quality of life, using the SF-12v2 Health Survey; and healthcare resource utilization.

**Results:**

4,876 workers reported being diagnosed with OA (45.0% mild, 45.9% moderate, and 9.1% severe); 34,896 workers comprised the non-OA comparator cohort. There was a greater proportion of females in the OA cohort (55.5% vs 45.6%; *P *< 0.0001) and more individuals in the 40-64 year and ≥ 65 year age ranges (*P *< 0.0001). As OA severity increased, workers reported more frequent pain, poorer quality of life, greater use of specific healthcare resources (hospitalizations) and reduced productivity. All outcomes indicated a significantly greater burden among workers with OA relative to those without OA (*P *< 0.0001). Estimated total annual costs per worker were $9,801 for mild OA, $14,761 for moderate OA, $22,111 for severe OA compared with $7,901 for workers without OA (*P *< 0.0001).

**Conclusions:**

Workers with OA were characterized by significant disease and economic burdens relative to workers without OA that substantially increased with greater self-rated OA severity. Greater levels of OA severity were associated with reductions in quality of life and productivity, and increases in healthcare resource utilization and costs.

## Background

Osteoarthritis (OA) ranks among the top causes of disability in the United States (US) [1] and is one of the leading causes of years of living with disability worldwide [2]. OA is also associated with substantial economic and societal burdens resulting from functional impairment, decreased quality of life, and increased healthcare resource utilization [3-8].

Although it has traditionally been considered a disease affecting an older population, OA has a profound effect on individuals who are still active participants in the workforce, often resulting in reduced productivity [8-12]. Despite the consistent reports of reduced productivity among workers with OA, data are still limited regarding the impact of OA among workers, and several studies have lumped OA with rheumatoid arthritis when evaluating employed populations [13-16]. One OA-specific study, which focused on absenteeism, made direct comparisons with a non-OA cohort [8].

However, presenteeism, generally defined as reduced productivity while at work, and suggested to be the primary source of lost productive time [17] was not examined. The Longitudinal Examination of Arthritis Pain (LEAP) study suggested that weekly fluctuations in OA pain were associated with changes in work absenteeism [18], and a more recent study suggested that OA-related pain has a profound impact on both absenteeism and presenteeism among employed individuals relative to those without OA pain [11]. However, the extent to which severity of OA as a condition may differentially affect outcomes among workers has not been previously considered.

Recent findings of significant relationships between patient self-rated OA severity and other outcomes, including pain, function, productivity, and costs in both US and European populations suggest that self-report of OA severity provides an accurate and tangible assessment of patients' perceptions of their disease [19-21]. patients' self-report of OA severity thus may be a useful approach to evaluate the impact and burden of OA in workers. The purpose of this study was to evaluate the impact of patient-rated OA severity on productivity and other outcomes including health-related quality of life (HRQoL), healthcare resource utilization, and costs in employed individuals relative to employed individuals without OA. Since both direct and indirect costs are evaluated, this study can be considered as taking the societal perspective.

## Methods

### Data source and population

Data were derived from the 2009 National Health and Wellness Survey (NHWS), a cross-sectional, self-administered, internet-based questionnaire administered annually to a nationwide sample of adults (≥ 18 years of age). The NHWS includes information on 75,000 individuals in the US and uses a random stratified sampling framework to ensure representativeness to the US population http://www.chsinternational.com/nhws.html. Comparisons of NHWS data with other sources (e.g. NHANES, NHIS) have been made elsewhere [22,23]. The NHWS was granted Institutional Review Board approval by Essex IRB (Lebanon, NJ; Protocol Number: CHS-NHWS-US2009-20045); all subjects provide informed consent prior to participation in the survey.

This analysis used data only for respondents ≥ 20 years of age and currently employed full-time, part-time, or self-employed. Workers meeting these criteria were categorized into four cohorts: mild OA, moderate OA, severe OA, and no OA. Workers in the OA groups were assigned based on self-reporting a diagnosis of OA ("Has your condition been diagnosed by a physician?", "yes" vs "no") and their responses to their severity of OA ("How severe is your arthritis?","mild" vs "moderate" vs "severe"). Subjects who did not report experiencing OA comprised the non-OA referent group.

### Outcomes

The demographic and health characteristics of the overall OA and non-OA cohorts were characterized and compared.

Subjects also reported on the presence of any pain and arthritis-related pain during the past 30 days, and rated their pain interference with normal work activities including work outside the home and housework ("not at all," "a little bit," "moderately" "quite a bit," and "extremely").

Work productivity was assessed using the Work Productivity and Activity Impairment (WPAI) scale [24]. The WPAI, not specific to OA, consists of four subscales that evaluate absenteeism, presenteeism, overall work impairment, and activity impairment during the previous seven days, generated in the form of percentages; higher values indicate greater impairment.

HRQoL was assessed using the physical (PCS) and mental component summary (MCS) scores from the self-reported SF-12v2 Health Survey [25], a validated measure for evaluating HRQoL. The PCS and MCS scores are normed to the US population (mean = 50, standard deviation = 10) and vary from 0 to 100; higher scores indicate better HRQoL. Health utility scores, calculated from the SF-6D and ranging from 0.29 to 1 provided a preference-based single index measure for health status [26].

Healthcare resource utilization within the past six months was self-reported by workers and included number of provider visits for both traditional healthcare (physician, emergency room [ER], and hospitalizations) and non-traditional healthcare e.g., acupuncturist, herbalist, etc.

Direct medical costs, which included physician visits, ER visits, and hospitalizations were estimated by multiplying the units of resource categories for six months by two to project annual number of visits, and then multiplying by the average cost of the resource derived from the Medical Expenditure Panel Survey database [27-29]. Indirect costs associated with lost productivity, regardless of causality, were calculated using the method of Lofland et al [30] based on data from the WPAI and median annual income values obtained through the Bureau of Labor Statistics (BLS) [31]. For each respondent, the percent overall work impairment (obtained from the WPAI) was multiplied by the annual income. Direct and indirect costs were summed to estimate total costs.

### Analyses

For categorical variables, chi-square tests were used, while analysis of variance (ANOVA) was used for continuous variables. A Bonferroni correction was applied to adjust for multiple comparisons; the resulting threshold for statistical significance is 0.001.

Analyses of quality of life were performed using multivariable models with the following demographic and clinical characteristics as covariates: age range (coded as 20-39 vs 40-64 and ≥ 65 years), gender, race/ethnicity (coded as non-Hispanic white, non-Hispanic black, Hispanic, or other), education (more than high school vs high school equivalent degree or less), income (< $25 K, $25 K to < $50 K, $50 K to < $75 K, ≥ $75 K, or decline to answer), Charlson Comorbidity Index [32] (CCI; dichotomized as 0 vs ≥ 1 because of the skewness of the distribution), health insurance (yes vs no), BMI (underweight [BMI < 18.5], normal [BMI 18.5 to < 25], overweight BMI 25 to < 30], obese [BMI ≥ 30], or decline to answer), employment (full-time, part-time, or self-employed), traditional healthcare visits (yes vs no), non-traditional healthcare visits (yes vs no), prescription drug use (yes vs no), ER visits (yes vs no), hospitalization (yes vs no) and experiencing pain in the past month (yes vs no).

Generalized linear models (GLMs) were fitted to predict work productivity, and activity impairment for the OA groups [33]. As work productivity impairment and activity impairment are often highly skewed, the GLMs specified a negative binomial distribution, testing whether adjusted log counts (controlling for covariates) differed across groups [34]. The multiplicative dispersion parameter was also added to adjust the standard errors to account for slight model under-dispersion, and antilogs of the regression estimates were calculated to yield rate ratios relative to no-OA [33]; rate ratios represent the x-fold difference in outcome relative to the referent. The overall effect of age on work productivity was evaluated from the individual regression analyses, and rate ratios were calculated for age categories of 40-64 years and ≥ 65 years relative to workers 20-39 years of age.

Determination and analysis of traditional healthcare resource utilization and direct costs were not adjusted for covariates; an analysis of indirect costs adjusting for the covariates used in the multivariable models was performed post-hoc. For non-traditional healthcare utilization, logistic regression was conducted to predict non-traditional healthcare provider visits (at least one visit vs none) based on OA severity and controlling for the covariates noted above. Analyses were run using SAS version 9.1 (SAS Institute Inc., Cary, NC, USA).

## Results

From the total US NHWS population, 33,765 respondents aged ≥ 20 years were not currently employed and were excluded from the analyses; of these excluded respondents, 13,178 (39.0%) were ≥ 65 years old. A total of 39,772 individuals met the inclusion criteria; 4,876 workers who reported being diagnosed with OA and 34,896 workers who served as the no-OA comparator cohort. Among workers with OA, severity was rated as mild, moderate, and severe by 45.0%, 45.9%, and 9.1% of individuals, respectively.

Demographic and clinical characteristics are shown in Table 1. The population was primarily non-Hispanic whites (66.2% to 75.2%), and overall, there was a greater proportion of females in the OA cohort (53.9% vs 45.6%)., Relative to workers without OA, workers with OA were characterized by greater proportions of individuals in the 40 to 64 year and ≥ 65 year age ranges, and by higher proportions of individuals meeting the BMI criteria of obesity (≥ 30 m^2^/kg).

**Table 1:** Weighted univariate statistics (to reflect the US population) for demographic characteristics of workers with osteoarthritis (OA) by self-reported severity category compared with employees without osteoarthritis

Variable			OA (n = 4,876)				Without OA (n = 34,896)	
	**Mild (n = 2,192)**		**Moderate (n = 2,240)**		**Severe (n = 444)**		**n**	**Weighted percent (SE)**

	**n**	**Weighted percent (SE)**	**n**	**Weighted percent (SE)**	**n**	**Weighted percent (SE)**		

Age range								

20-39 years	338	18.6 (0.9)	298	16.1 (0.9)	49	13.0 (1.8)	15,705	49.7 (0.3)

40-64 years	1,339	65.0 (1.1)	1,447	68.8 (1.1)	312	74.0 (2.7)	16,926	46.6 (0.3)

≥ 65 years	515	16.42 (0.9)	495	15.1 (0.9)	83	13.0 (2.5)	2,265	3.7 (0.1)

Gender								

Male	1,120	49.2 (1.2)	1,008	44.1 (1.2)	190	41.7 (2.6)	18,460	54.4 (0.3)

Female	1,072	50.8 (1.2)	1,232	56.0 (1.2)	254	58.4 (2.6)	16,436	45.6 (0.3)

Race/ethnicity								

White, non- Hispanic	1,765	75.2 (1.1)	1,757	73.1 (1.1)	338	69.7 (2.9)	24,279	66.2 (0.3)

Black, non- Hispanic	174	8.8 (0.7)	223	10.6 (0.8)	63	16.5 (2.6)	4,143	12.1 (0.2)

Hispanic white	68	5.8 (0.7)	80	6.6 (0.7)	18	6.6 (1.5)	1,959	8.6 (0.2)

Hispanic black	3	0.3 (0.1)	7	0.6 (0.2)	2	0.6 (0.4)	148	0.7 (0.1)

Other	182	9.9 (0.7)	173	9.2 (0.7)	23	6.6 (1.4)	4,367	12.5(0.2)

Education								

≤ High school graduate	312	15.1 (0.9)	385	17.4 (0.9)	99	22.5 (2.2)	5,791	17.0 (0.2)

> High school	1,880	84.9 (0.9)	1,855	82.7 (0.9)	345	77.5 (2.2)	29,104	83.0 (0.2)

Employment								

Full time	1,297	61.2 (1.1)	1,282	59.5 (1.2)	236	53.3 (2.8)	24,473	71.3 (0.3)

Part time	557	23.3 (1.0)	586	24.8 (1.1)	106	24.3 (2.7)	6,442	17.9 (0.2)

Self- employed	338	15.5 (0.9)	372	15.7 (0.9)	102	22.5 (2.1)	3,981	10.8 ± 0.2)

Income								

< $25,000	213	9.6 (0.7)	291	13.4 (0.8)	89	20.0 (2.1)	3,906	11.7 (0.2)

$25,000 to $49,999	595	27.1 (1.0)	703	31.4 (1.1)	131	28.6 (2.3)	10,045	29.2 (0.3)

$50,000 to $74,999	560	25.7 (1.0)	531	23.8 (1.0)	90	20.2 (2.0)	8,461	24.2 (0.2)

≥ $75,000	711	31.8 (1.1)	619	27.6 (1.0)	108	25.2 (2.7)	10,971	30.8 (0.3)

Declined to answer	113	5.8 (0.6)	96	3.9 (0.4)	26	6.0 (1.2)	1,513	4.2 (0.1)

Health insurance								

Yes	1,955	88.2 (0.8)	1,951	86.0 (0.8)	386	86.0 (1.8)	28,731	81.3 (0.2)

No	237	11.8 (0.8)	289	14.0 (0.8)	58	14.0 (1.8)	6,165	18.7 (0.2)

BMI (m^2^/kg)								

Underweight(< 18.5)	19	1.0 (0.3)	22	0.8 (0.2)	3	0.7 (0.4)	604	1.8 (0.1)

Normal(18.5 to < 25)	527	25.0 (1.0)	431	20.2 (1.0)	74	18.7 (2.7)	11,273	32.6 (0.3)

Overweight (25 to < 30)	726	32.9 (1.1)	722	31.9 (1.1)	130	27.9 (2.3)	11,761	33.6 (0.3)

Obese (≥ 30)	890	39.8 (1.1)	1,027	45.3 (1.2)	229	51.0 (2.7)	10,635	30.2 (0.3)

Declined to answer	30	1.3 (0.3)	38	1.7 (0.3)	8	1.7 (0.6)	623	1.8 (0.1)

Charlson Comorbidity Index (CCI)								

CCI of 0	1,437	67.2 (1.1)	1,296	59.1 (1.1)	213	47.7 (2.7)	28,294	82.1 (0.2)

CCI of 1 or more	755	32.8 (1.1)	944	40.9 (1.1)	231	52.6 (2.7)	6,602	17.9 (0.2)

**Figure 1:**
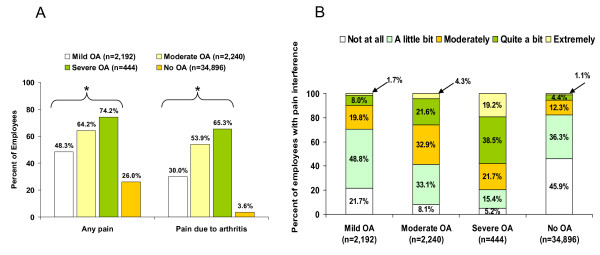
**Pain and pain-related interference among workers with osteoarthritis (OA) by self-rated OA severity and workers without OA**. A) Proportion of workers reporting any pain and arthritis-related pain. *p < 0.0001 across severity levels vs no OA. B) Proportion of workers reporting pain interference with normal work activities including work outside the home and housework. p < 0.0001 across severity levels vs no OA for each category of pain interference.

The proportion of workers with OA reporting any pain and arthritis-related pain during the past 30 days increased at higher self-rated OA severity levels (Figure 1A). The overall effect for both pain categories was significant relative to the non-OA workers (both p < 0.0001) (Figure 1A). Significantly higher proportions of workers with OA reported greater pain interference with daily activities including work outside the home and housework relative to non-OA workers (Figure 1B; p < 0.0001). As OA severity increased, the proportion of workers reporting greater levels of pain interference also increased.

**Figure 2:**
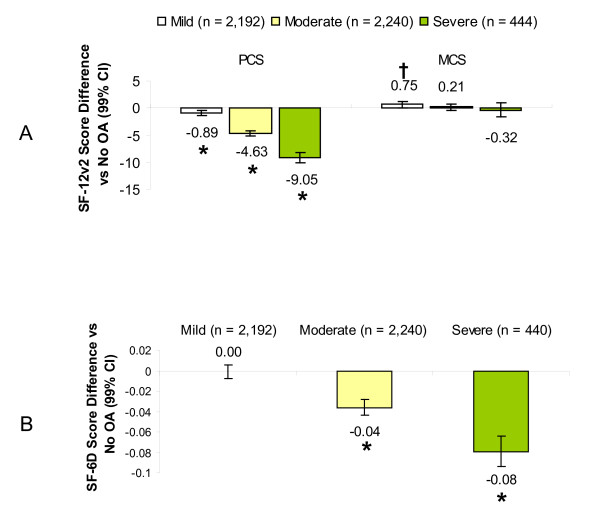
**Impact of osteoarthritis (OA) among workers, by self-rated OA severity, on health-related quality of life relative to workers without OA**. Values represent adjusted differences in scores among workers with each level of OA severity relative to workers without OA for the Physical (PCS) and Mental (MCS) Component summary scores on the SF-12v2 (A) and the SF-6 health utility index (B). Negative values represent poorer health-related quality of life. *p < 0.0001 and ^†^p < 0.001 relative to workers without OA.

Workers with mild, moderate and severe OA reported significantly worsening adjusted SF-12v2 PCS scores as severity level increased (p < 0.0001), and for MCS, there was a significant increase in the mild OA group compared with non-OA workers (Figure 2A). After controlling for covariates, workers with moderate and severe OA reported health utility scores significantly lower, by 0.04 and 0.08 points, respectively, relative to workers without OA (Figure 2B); there was no difference between mild OA and no OA.

**Figure 3:**
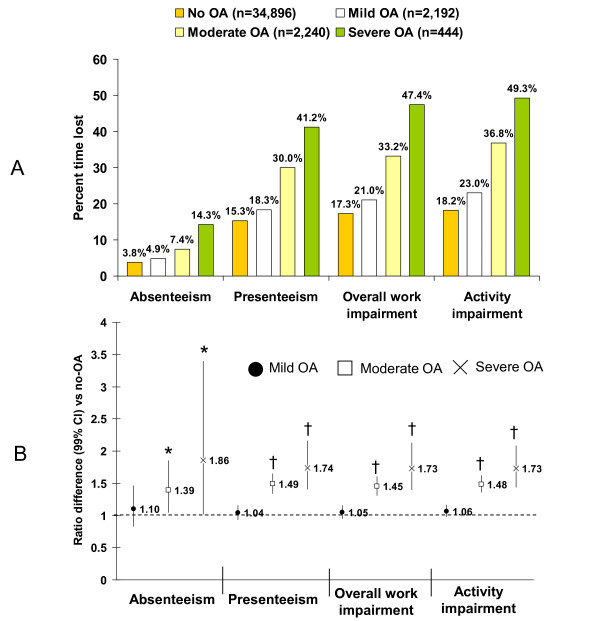
**Lost productivity as measured by the Work Productivity and Activity Impairment questionnaire (WPAI) **[22]. A) Percent time lost in the past week (unadjusted). B) Adjusted rate ratios for work and activity impairment among workers workers by self-rated OA severity relative to workers without OA. *p < 0.001 and ^†^p < 0.0001 relative to workers without OA.

Work and activity impairment (Figure 3A) showed consistently greater impairment among workers with OA relative to those without OA; impairment increased with increasing OA severity. Lost productivity due to presenteeism was approximately 3-4 times greater than that due to absenteeism across all cohorts even among workers without OA. Overall, workers with OA decreased their productivity by approximately one-third; those with severe OA had almost a 50% reduction in work time. The adjusted rate ratio (Figure 3B) indicates a difference in magnitude of impairment of 1.39 for absenteeism in the moderate OA group (i.e. 39% more absenteeism than workers without OA). Workers with moderate and severe OA reported significantly higher percentages of absenteeism, presenteeism, overall work impairment, and activity impairment relative to workers without OA (p < 0.001); no differences were found for the mild OA group, since the 99% CI crosses 1, indicating lack of evidence for differences in productivity among workers with mild OA relative to workers with no OA. In terms of hours lost, workers with mild, moderate, and severe OA lost a mean ± standard deviation of 1.6 ± 5.7, 2.5 ± 7.1, and 4.8 ± 9.7 h, respectively, relative to workers with no OA (1.3 ± 5.6 h; overall p < 0.0001). Similarly, hours lost due to presenteeism were 5.86 ± 8.1 for mild OA, 9.37 ± 9.6 for moderate OA, and 12.5 ± 12.0 for severe OA relative to workers without OA (5.05 ± 8.5; overall p < 0.0001).

Evaluation of the impact of age on productivity among workers with OA (Table 2) showed that work productivity loss was greater in younger workers. Relative to workers 20-39 years of age, workers in the 40-64-year age group had approximately 1.4 times less absenteeism, presenteeism, and overall work impairment. Similarly, workers ≥ 65 years had 2.7, 2.5, and 2.4 times less absenteeism, presenteeism, and overall work impairment, respectively. As worker age increased, so did the proportion of self-employed individuals, from 7.5% in those 20-39 years of age, to 13.3% and 29% in the middle and the older age groups. Similarly, there appeared to be an increase in the proportion of individuals employed part-time in the older age group (43.1%) relative to the middle-age (16.43%) and younger (18.2%) age groups.

**Table 2:** Effect of age on work and productivity impairment among workers with osteoarthritis.

WPAI Question	40-64 years of age (n = 1,339)		≥ 65 years of age (n = 515)	
	**Rate ratio (95% CI)**	**p-value**	**Rate ratio (95% CI)**	**p-value**

Absenteeism	0.707 (0.614, 0.815)	< 0.0001	0.376 (0.289, 0.489)	< 0.0001

Presenteeism	0.733 (0.698, 0.769)	< 0.0001	0.406 (0.369, 0.445)	< 0.0001

Overall work impairment	0.737 (0.702, 0.773)	< 0.0001	0.421 (0.384, 0.462)	< 0.0001

Activity impairment	0.778 (0.745, 0811)	< 0.0001	0.512 (0.472, 0.555)	< 0.0001

Rate ratios, adjusted for other covariates, are relative to the referent of workers 20-39 years of age (n = 338). A rate ratio < 1 indicates a reduction relative to the referent, and the reciprocal of the rate ratio indicates the magnitude of the reduction

Unadjusted traditional healthcare visits over the prior 6 months were significantly greater among workers with OA relative to no OA (p < 0.0001); the proportion of workers with these visits were generally similar across OA severity levels (Table 3). There was significantly greater use of ER visits and hospitalizations with increasing OA severity (p < 0.0001). Although with increasing OA severity there were slight increases in the proportion of subjects using non-traditional healthcare resources (Table 3), logistic regression analysis showed that non-traditional resource utilization decreased at higher OA severity levels relative to the non-OA cohort. The calculated odds ratios for a visit to a non-traditional healthcare provider showed that subjects with mild OA were 1.25 times more likely (99% CI 1.09, 1.43) to visit a non-traditional healthcare provider than workers without OA (p = 0.0007), although there were no significant differences between the moderate and severe OA workers relative to non-OA workers; the calculated odds ratios were 1.12 (99% CI 0.97, 1.28) and 0.92 (99% CI 0.69, 1.22) for moderate and severe OA, respectively. The mean number of prescriptions over the prior 6 months increased at greater levels of OA severity, and was significantly greater than workers without OA (Table 3)

**Table 3:** Unadjusted 6-month healthcare resource utilization among workers with osteoarthritis (OA) by self-reported severity category compared with workers without osteoarthritis

Health Resource Category	OA (n = 4,876)			Without OA (n = 34,896)	p-value (OA vs no OA)
	**Mild (n = 2,192)**	**Moderate(n = 2,240)**	**Severe (n = 444)**		

Traditional healthcare visits, weighted percent (SE)	88.3 (0.7)	90.0 (0.7)	89.34 (1.6)	71.7 (0.3)	< 0.0001

Emergency room visits, weighted percent (SE)	13.2 (0.8)	18.3 (1.0)	28.36 (2.8)	10.2 (0.2)	< 0.0001

Hospitalizations, weighted percent (SE)	8.5 (0.7)	11.1 (0.8)	20.9 (2.1)	5.1 (0.1)	< 0.0001

Non traditional healthcare visits, weighted percent (SE)	30.2 (1.1)	31.9 (1.1)	34.3 (2.8)	20.1 (0.2)	< 0.0001

Number of prescriptions, mean ± standard deviation	3.7 ± 4.1	5.0 ± 4.8	6.4 ± 5.5	2.0 ± 3.2	< 0.0001

**Figure 4:**
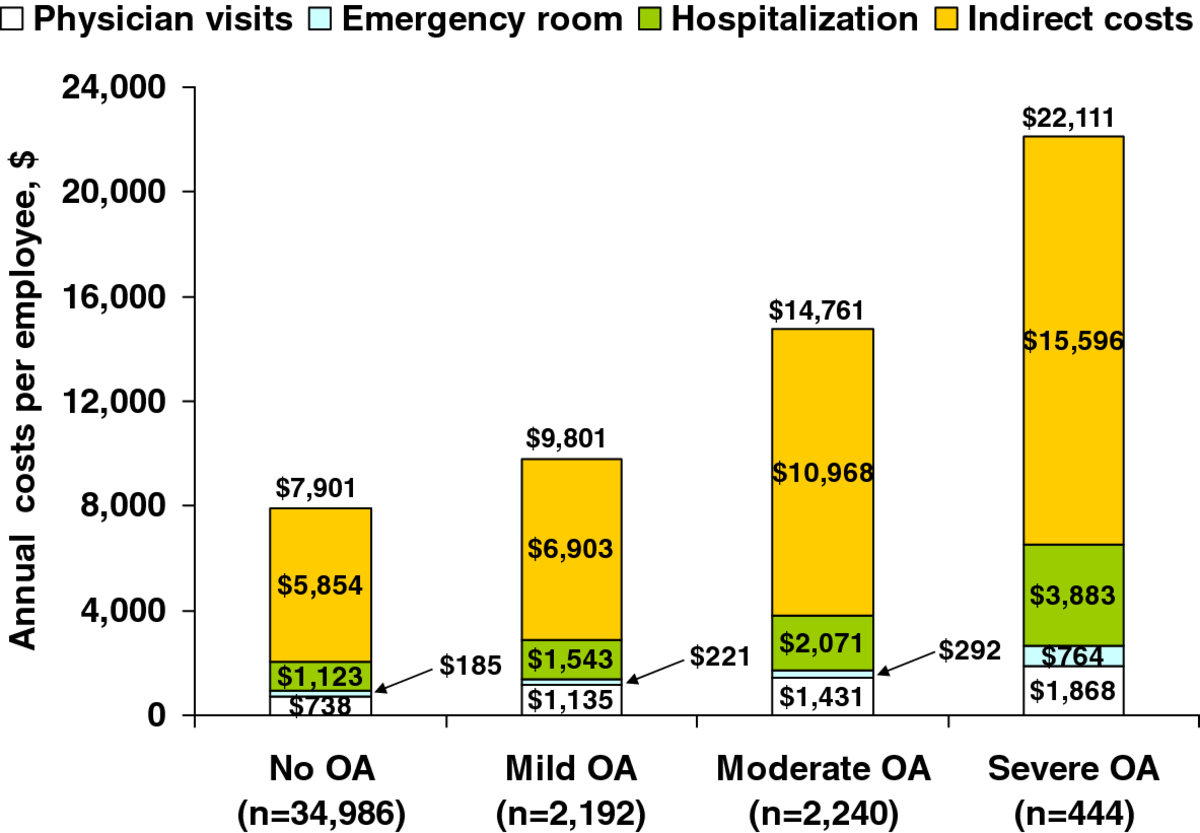
**Annual unadjusted costs per individual among workers with osteoarthritis (OA) by self-rated OA severity and workers without OA**. Overall p < 0.0001 for each cost category relative to workers with no OA.

Estimated unadjusted annual total costs per worker (Figure 4) were $9,801 for mild OA, $14,761 for moderate OA, and $22,111 for severe OA, compared with $7,901 per worker without OA (p < 0.0001). Costs were higher with increasing OA severity, and were significantly higher relative to workers without OA (p < 0.0001) (Figure 4). Indirect costs, based on lost productivity, were the primary driver of costs, accounting for 70%-74% of total costs, even among workers without OA. In workers with moderate and severe OA, the unadjusted indirect costs of $10,968 and $15,596, respectively, were 2- and 3-fold higher than workers without OA ($5,854). When indirect costs were adjusted for covariates in the multivariable models, these costs were lower than unadjusted costs. However, the adjusted indirect costs were significantly greater (p < 0.0001) among patients with moderate ($7,413; 99% CI $6,734, $8,161) and severe OA ($8,852; 99% CI $7,177, $10,918) relative to no-OA ($5,140; 99% CI $5,021, $5,262), although there was no difference between no-OA and mild OA ($5,392; 99% CI $4,905, $5,927).

## Discussion

Self-report of OA severity has been shown to be accurate and relevant in clinical practice [19-21]. This study provides a practical application of using self-report as an indicator of OA severity and demonstrates that as self-rated OA severity increases, there is a greater burden relative to workers without OA.

Many of the demographic differences that were identified between the OA and non-OA cohorts are consistent with what may be expected regarding the epidemiology and risk factors for OA (older, female, non-Hispanic white, greater comorbidities, tendency toward obesity). These variables were included as covariates in the multivariable analyses of quality of life and productivity, and thus the observed differences in these outcomes were likely related to the presence of OA.

Since epidemiologic data on individuals with OA younger than 45 years is sparse, it is interesting to note that the prevalence of OA was 4.2% for all workers 20-39 years of age, and that among workers with OA, 16.9% were in this age group. Additionally, approximately half of the workers (50.7%) with OA in this age group rated their OA as at least moderate severity. These data indicate that OA is likely to be more prevalent and have a greater impact in a younger population than has previously been thought based on the consideration of OA primarily as an age-related disease.

Workers with OA reported significantly lower health status relative to non-OA workers as measured by SF-6D utility values. The observed differences of -0.04 points and -0.08 points for moderate and severe OA relative to non-OA, respectively, were clinically significant; differences of at least 0.03 points are proposed to be clinically meaningful [35]. Pairwise differences in health status among OA severity levels also exceeded 0.03, suggesting clinical significance. This trend of poorer health with increasing self-rated OA severity is consistent with previous observations using the EuroQol (EQ-5D) health index in US and European OA populations [20,21].

OA affects physical functioning, and it is therefore not surprising that effects were greater on physical components (PCS) of HRQoL than on mental components (MCS). Since differences of greater than 3 points between groups are considered clinically significant [36], the differences in PCS scores were clinically meaningful as well as statistically significant. In contrast, there was little change in the MCS, and although the score among workers with mild OA was statistically higher than among workers without OA, this is likely due to measurement error, possibly because of the large population. Of note, both the PCS and MCS scores are normed to the US population, enhancing generalizability.

The frequency of general pain and arthritis pain was significantly greater among workers with OA relative to those without, and higher at increasing levels of OA severity. Although OA pain and its treatment are associated with reduced productivity and increased costs [11,37], it remains to be determined whether these effects are related to pain severity, frequency, or both. Pain is likely to be only one of several factors that contribute to patients' perceptions of OA severity, and while experiencing pain in the past month was included as a covariate, pain severity was not. Nevertheless, the pain-related interference, which increased with greater OA severity and was significantly higher than non-OA workers, is consistent with an association between OA severity and specific activities of daily living [19], and provides a foundation for the potential impact of OA severity on work impairment.

Workers with OA were characterized by significantly greater work and activity impairment relative to those without OA, and the separation between cohorts increased with greater OA severity. Among workers with moderate and severe OA, approximately one-third (33.2%) and one-half (47.4%) of worker productivity was lost, respectively, compared with 17.3% among non-OA workers. While several studies evaluated OA-related absenteeism [8-10,18], few data exist on presenteeism, despite presenteeism being suggested as the primary source of lost productivity in the general arthritis population [13,16,17,38]. This study confirms presenteeism as the primary source of work impairment in workers with OA, and characterizes the magnitude of this impairment as 3-4 times greater than that due to absenteeism, resulting in loss of more than a day's work/week among workers with moderate and severe OA.

Interestingly, lost productivity decreased with increasing age among workers with OA. The reasons underlying this observation cannot be established based on the data from this study, and few other studies have evaluated OA and its impact across these age groups. Nevertheless, several suggestions may be proposed to account for these data. First, it is possible that at least some of the differences in lost productivity between the younger and older age groups may be explained by the concomitant increase in self-employment and part-time work observed with increasing age. These types of employment, especially the former, may potentially allow workers to benefit from a more flexible work environment, thereby reducing lost productivity. In this regard, it should also be noted that while all individuals were employed at the time of the survey, no information was available on whether workers switched jobs, received accommodations at work for OA disability, or transitioned out and back into the workforce over time. Such workforce transitions have previously been shown to be common among workers with arthritis [14]. Second, coping strategies and self-efficacy, which are prognostic factors for outcomes in individuals with OA [39], are likely to be different among the age groups [40]. Such strategies may also relate to duration of disease, which was not captured in the current study.

The consequences of lost productivity were profound as manifested by their impact on costs. Although indirect costs were the primary cost driver, including among workers without OA, the magnitude of work impairment was especially apparent among workers with moderate and severe OA, resulting in unadjusted indirect costs that were 87% and 166% higher, respectively, than among workers without OA, and adjusted indirect costs 44% and 72% higher, respectively.

The magnitude of the unadjusted indirect costs ($6,903, $10.968, and $15,596 for mild, moderate, and severe OA, respectively) is consistent with estimates for employees with mild ($6,096), moderate ($13,251), and severe ($17,214) self-rated OA in a clinical practice-derived database [19]. However, they are in contrast to other studies that reported low indirect costs [9,37,41]. Since there are no standardized methods for estimating indirect costs in OA [42], these discrepancies may be attributed to differences in populations or methodologies. It is likely that inadequately accounting for presenteeism in other studies contributed to underestimation of costs. Differences in methodology may also account for the observation that our estimated mean medical costs of workers with OA ($4,403 across all severity categories) were lower than in other recently reported studies ($6,984-$8,201) [9,10].

The data additionally show that more workers with OA reported use of traditional healthcare, and had associated higher costs across categories than the non-OA workers. The primary driver of direct costs was hospitalizations, likely due to the high cost per event. Although workers with OA were prescribed significantly more medications than those without OA, this was not included in the costs. Furthermore, whether these visits and medications were specifically related to OA could not be ascertained.

It is worth noting that after adjusting for covariates, non-traditional health provider visits (e.g. acupuncturists, herbalists, etc.) were used by a significantly greater proportion of individuals only among workers with mild OA relative to workers without OA. There is scarce information on utilization and outcomes of non-traditional provider care for OA, possibly because these visits are not generally included in insurance plans or claims databases.

It should be recognized that in the clinical setting, a variety of covariates are likely to impact healthcare-seeking behavior by patients, management strategies by providers, and work-loss-related compensation among employers. However, the resource utilization and total cost analyses were not adjusted for covariates since we wanted to provide a more complete perspective of the burden among these workers; in particular, unadjusted costs are often used to characterize the overall cost burden. Additionally, our multivariable models contained covariates that were outcomes in the resource utilization and costs analysis. Consequently, as a result of using unadjusted values, a conservative approach should be used for interpreting the implications of these analyses.

Strengths of this study include our ability to capture a wide variety of outcomes and the focus on an employed population, since disease burden in an active workforce is of economic importance to a variety of stakeholders. The large sample size and use of population-level analyses based on weighted assessments to reflect the demographic composition of the US population are additional strengths that enhance the generalizability of this study. Conversely, the large sample size may also be considered a limitation, since it is likely that some of the statistical significance could be ascribed to the large population.

Additional limitations include the use of self-report and that the OA diagnosis was not clinically confirmed. The latter could potentially introduce selection bias, since it is possible that workers in both cohorts may not have been clear about whether they had the correct diagnosis for inclusion/exclusion in their respective cohorts. However, given the large sample size, it is likely that the number of workers inappropriately placed in the cohorts would not substantially bias the results. Furthermore, this potential for bias does not preclude patient report as an important resource for evaluating outcomes, especially related to productivity.

Since the WPAI was not OA specific, the observed relationships between OA and productivity should be considered associative rather than causal. Similarly, the higher resource utilization and costs cannot be ascribed specifically to OA, since there are no claims linking resource use with the disease and symptoms of interest. Nevertheless, lost productivity in workers with OA was higher with increasing OA severity, and resulted in significantly greater indirect costs among workers with OA that may be of special concern to employers.

Although information was obtained on salary ranges and education, the type of employment or worker occupation was not considered. Type of employment is not only a risk factor for OA resulting from specific occupational activities [43,44], but is also likely to affect productivity, since workers with OA may be likely to avoid certain work activities. A similar limitation is that the joint affected was not determined; specific sites of OA (lower back, neck and knee) have been shown to be predictors of greater productivity losses [45] and may also differentially affect healthcare resource utilization and associated costs. It is important to recognize that these factors may have implications for management strategies, and their absence in our analysis may reduce the generalizability of the results.

Although the number of prescribed drugs was captured by patient report, this information could not be used to estimate pharmacotherapy costs, since the source of our data was not a medical claims database. Thus, direct costs are likely to be underestimated. Furthermore, derivation of annual costs was based on extrapolation of 6-month data to 1 year, and may not adequately reflect annual resource utilization.

## Conclusions

Workers with OA had significantly lower productivity and HRQoL, and significantly higher healthcare resource utilization and costs than non-OA workers. Increased OA severity was associated with incremental productivity losses and costs, relative to workers with less severe OA and to those without OA. Total costs were driven by indirect costs resulting from lower productivity, with presenteeism accounting for the greatest proportion of lost work time. These results illustrate a practical application of asking patients to self-rate their OA severity. The association of these ratings with a variety of outcomes suggests the utility of asking patients to self-rate their disease in clinical practice to provide greater relevancy from the patient's perspective.

The contribution of OA severity to the patient and economic burdens of this disease was also demonstrated in this study. Such information may be useful to employers and healthcare providers when considering management strategies and workplace accommodations to improve productivity and other outcomes that can help alleviate the burden of OA. The results also suggest that there is a need for additional studies that can stratify by specific sites of OA, as well as by different occupations, and more clearly determine what proportion of the higher resource utilization and costs may be attributable specifically to OA. Studies are also warranted to further evaluate and quantify the direct costs associated with different levels of OA severity.

## Abbreviations

BMI: Body mass index; ER: Emergency room; HRQoL: Health-related quality of life; MCS: Mental component summary of the SF-12v2 Health Survey; NHWS: National Health and Wellness Survey; OA: Osteoarthritis; PCS: Physical component summary of the SF-12v2 Health Survey; WPAI: Work Productivity and Impairment scale.

## Competing interests

Alesia Sadosky and Margaret McDonald are employees and stockholders of Pfizer Inc, the sponsor of this study. Dan Pettitt was a Pfizer employee during the conduct of this study. Marco DiBonaventura and Shaloo Gupta are employees of Kantar Health, who conducted the National Health and Wellness Survey and analyzed the data on behalf of Pfizer Inc. Dr Silverman is a consultant to Pfizer and Lilly. Dr. Silverman did not receive financial support for this project.

## Authors' contributions

All authors contributed to the study design, statistical analysis plan, results interpretation, and review of the draft manuscript; the final manuscript was read and approved by all authors.
